# Cost-Effectiveness of Tdap Vaccination of Adults Aged ≥65 Years in the Prevention of Pertussis in the US: A Dynamic Model of Disease Transmission

**DOI:** 10.1371/journal.pone.0072723

**Published:** 2014-01-09

**Authors:** Lisa J. McGarry, Girishanthy Krishnarajah, Gregory Hill, Cristina Masseria, Michelle Skornicki, Narin Pruttivarasin, Bhakti Arondekar, Julie Roiz, Stephen I. Pelton, Milton C. Weinstein

**Affiliations:** 1 OptumInsight, Cambridge, Massachusetts, United States of America; 2 GlaxoSmithKline, Philadelphia, Pennsylvania, United States of America; 3 Boston University, Boston, Massachusetts, United States of America; 4 Harvard University, Harvard School of Public Health, Boston, Massachusetts, United States of America; Old Dominion University, United States of America

## Abstract

**Objectives:**

In February 2012, the Advisory Committee on Immunization Practices (ACIP) advised that all adults aged ≥65 years receive a single dose of reduced-antigen-content tetanus, diphtheria, and acellular pertussis (Tdap), expanding on a 2010 recommendation for adults >65 that was limited to those with close contact with infants. We evaluated clinical and economic outcomes of adding Tdap booster of adults aged ≥65 to “baseline” practice [full-strength DTaP administered from 2 months to 4–6 years, and one dose of Tdap at 11–64 years replacing decennial Td booster], using a dynamic model.

**Methods:**

We constructed a population-level disease transmission model to evaluate the cost-effectiveness of supplementing baseline practice by vaccinating 10% of eligible adults aged ≥65 with Tdap replacing the decennial Td booster. US population effects, including indirect benefits accrued by unvaccinated persons, were estimated during a 1-year period after disease incidence reached a new steady state, with consequences of deaths and long-term pertussis sequelae projected over remaining lifetimes. Model outputs include: cases by severity, encephalopathy, deaths, costs (of vaccination and pertussis care) and quality-adjusted life-years (QALYs) associated with each strategy. Results in terms of incremental cost/QALY gained are presented from payer and societal perspectives. Sensitivity analyses vary key parameters within plausible ranges.

**Results:**

For the US population, the intervention is expected to prevent >97,000 cases (>4,000 severe and >5,000 among infants) of pertussis annually at steady state. Additional vaccination costs are $4.7 million. Net cost savings, including vaccination costs, are $47.7 million (societal perspective) and $44.8 million (payer perspective). From both perspectives, the intervention strategy is dominant (less costly, and more effective by >3,000 QALYs) versus baseline. Results are robust to sensitivity analyses and alternative scenarios.

**Conclusions:**

Immunization of eligible adults aged ≥65, consistent with the current ACIP recommendation, is cost saving from both payer and societal perspectives.

## Introduction

The full-strength diphtheria-tetanus-acellular pertussis vaccine, DTaP, was first approved in the US in 1991 for the 4^th^ and 5^th^ doses in the 5-dose diphtheria, tetanus and pertussis series. The vaccine received approval for all 5 doses in 1997 [1]. The Advisory Committee on Immunization Practices (ACIP) recommends that children receive 5 doses of DTaP, at ages 2, 4, 6, 15–18 months, and 4–6 years. The tetanus-diphtheria vaccine (Td) is given to adolescents and adults as a booster every 10 years, or after an exposure to tetanus. As of 2005, ACIP recommends that adolescents 11–18 years (preferably at age 11 or 12) and adults 19 through 64 years receive a single dose of reduced-antigen content combined tetanus, diphtheria, and pertussis (Tdap) vaccine, which also protects against pertussis, as a one-time replacement for a Td decennial booster [2]. In 2010, ACIP recommended the expanded use of Tdap, advising that children aged 7 through 10 years not previously fully vaccinated against pertussis receive a single dose of Tdap, and that adults ≥65 years who have close contact with an infant receive a single dose of Tdap if they have not previously received the booster [3]. Despite recommendations, Tdap coverage remained incomplete in 2011, at 78.2% for the adolescent booster and 8.2% for the decennial booster among adults aged <65.years [4] In February 2012, ACIP extended the recommendation of Tdap boosting to all adults (including those aged ≥65 years) without regard to interval since last tetanus or diphtheria toxoid-containing vaccine [5].

After the introduction of adult and adolescent Tdap vaccination in 2005, pertussis incidence decreased from 2004 levels. However, since 2007, incidence has been increasing and reached its most recent peak in 2010 [6]. Pertussis incidence continues to be higher than in the 1990s, and outbreaks have occurred, most recently in California and Washington State [7], [8]. It is unclear whether this recent increase in incidence reflects changes in epidemiology, perhaps due to faster than expected waning of the acellular pertussis vaccine [9], or to changes in disease reporting. Although pertussis is nationally notifiable, many cases are believed to go unrecognized and/or unreported [10], so the true incidence of disease is uncertain.

Vaccines such as Tdap are unusual among health-care interventions in that their benefits may accrue both to those receiving the vaccination and others who are not vaccine-protected. This is most apparent when vaccination leads to disease eradication such as was the case for smallpox, or when a once common and devastating disease such as polio is controlled through an ongoing mass vaccination program [11]. Although the incidence of pertussis is reduced from the pre-vaccine levels, outbreaks still occur among unprotected and partially protected populations, including infants aged less than 6 months, among whom the death rate is higher than among other age groups [12], [13]. Because these vulnerable populations cannot be directly protected through vaccination, they rely on indirect protection from vaccination in other age groups with which they come into contact (“cocooning”).

Studies have provided evidence that administering a pertussis booster in previously-vaccinated populations is cost-effective [14]–[16] and that cocooning by vaccinating close contacts of infants is also a cost-effective way to prevent adverse outcomes in infants [17]. The current analysis assesses the impact of the 2010 and 2012 decisions to add Tdap vaccination in adults aged ≥65 years to the baseline practice prior to 2010. We used a dynamic transmission model to capture both direct effects of vaccination among those vaccinated as well as indirect effects of vaccination in the larger population due to reductions in circulating pertussis. Direct effects of the ≥65 vaccination program versus baseline practice have been documented in a prior study [18] that considered only vaccine efficacy among vaccinated persons.The current study was designed to examine both the direct and hypothetical indirect effects of vaccination based on assumptions regarding population mixing and disease transmission. We present results from both a societal and a payer perspective.

## Methods

This study used a population-level disease-transmission model programmed in Microsoft Excel using Visual Basic for Applications (VBA) to assess public health and cost implications of two alternative vaccination strategies. (1) ***The baseline vaccination strategy*** is the vaccination practice prior to the updated 2010 ACIP recommendations, of administering DTaP from age 2 months to 4–6 years, and one dose of Tdap once to individuals 11–64 years of age in place of the decennial Td booster. (2) ***The intervention strategy*** is Tdap vaccination of adults aged 65 years in addition to the baseline vaccination strategy, consistent with the 2010 and 2012 recommendations.

We estimated the effects of the intervention strategy during a single 1-year season, with consequences of events occurring during this year projected over the lifetimes of individuals who die or suffer long-term sequelae. The analytic horizon was selected to reflect the seasonal nature of pertussis. We assumed that each of the vaccination strategies had been fully implemented and that disease incidence rates had reached equilibrium by the study year such that, with no change in intervention, each year thereafter could be assumed to have the same incidence.

### Epidemiologic model

**Figure 1:**
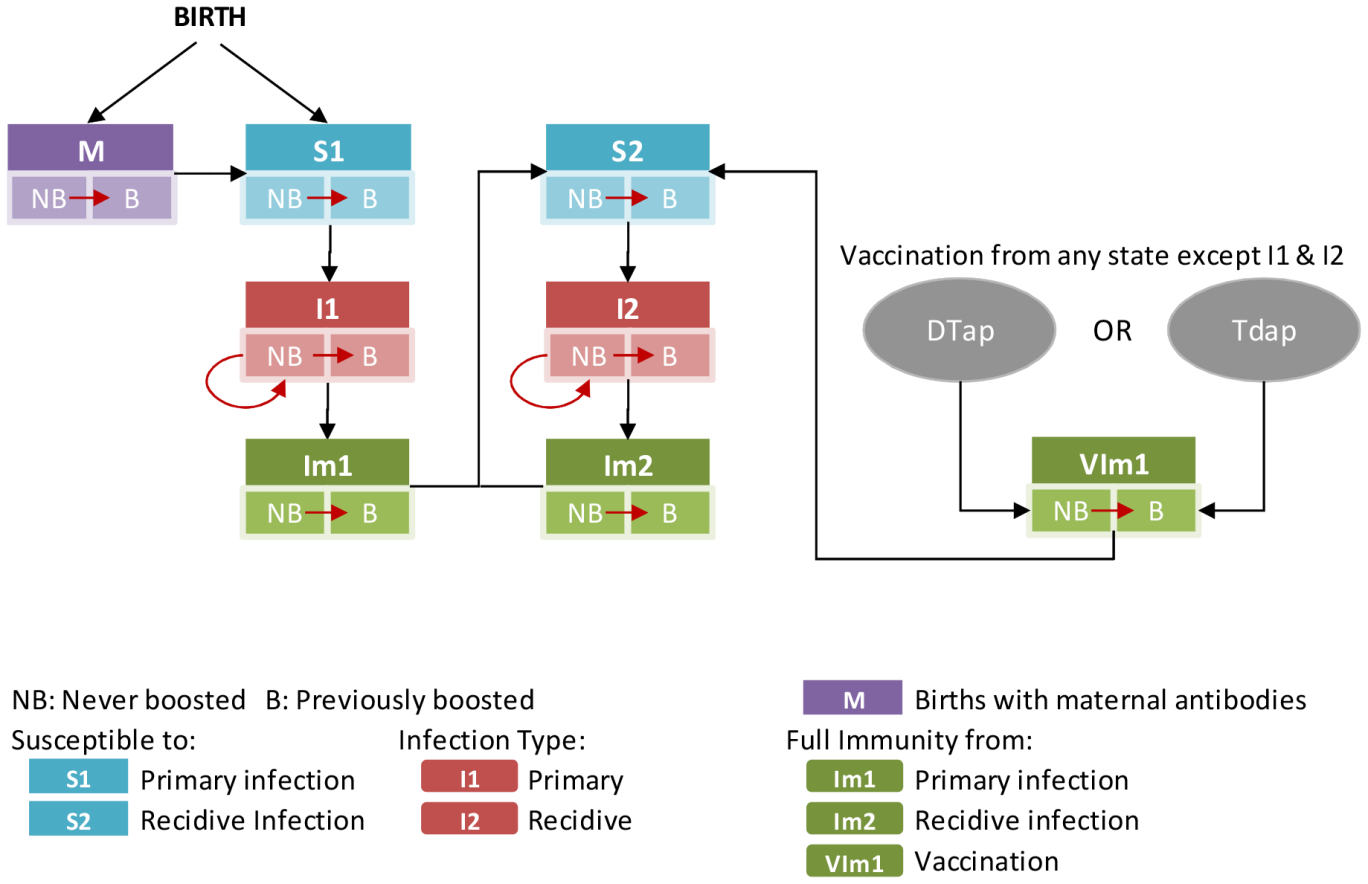
Transmission Model.

The epidemiologic analysis was conducted using a dynamic transmission model based on a Tdap cost-effectiveness model developed by the University of Groningen (Netherlands) [14], composed of 8 compartments representing mutually exclusive health states. The model is age-structured with compartments repeated for each month of age below 1 year and 1-year age groups from 1 to 99 years old (Figure 1).

Two types of infection were modeled: primary (I1) and recidive (I2). Primary infection is defined as the first infection with pertussis among individuals who have not previously been vaccinated or infected; recidive is infection with pertussis among those previously vaccinated or infected but no longer immune. Time spent in each infected compartment corresponds to the “infectious” period, during which transmission may occur, and differs by infection type.

Infants may begin the model in the “M” compartment, where they are protected by maternal antibodies, or in the susceptible “S1” compartment. When maternal immunity wanes, those initially protected become susceptible to primary pertussis infection. Individuals of all ages in S1 can acquire infection from contact with an infectious person, and move to primary infection. When patients recover from primary infection, they initially have immunity against pertussis. As this immunity wanes, they become susceptible (“S2”) to a recidive infection (“I2”).The waning from immunity to susceptibility follows the same pathway for recidive as for primary infection.

Individuals effectively vaccinated against pertussis are protected by vaccination (“VIm1”). Upon waning, they become susceptible to recidive infection in the same way as those recovering from infection. Vaccination is assumed to provide no additional protection to infected individuals beyond the immunity already conferred by the infection. Each compartment is subdivided to track whether individuals have received Tdap vaccination. Individuals previously vaccinated with Tdap are indicated by “B” (boosted), and those not previously vaccinated with Tdap are labeled “NB”. Consistent with current recommendations, individuals may receive Tdap only once, although individuals previously boosted may no longer be protected.

Transmission rates within the population depend on the ages of susceptible and infected individuals. Both the transmission rates and duration of infectiousness differ depending on the type of infections, with recidive infection (I2) assumed to be 70% as transmissible as a primary infection [14]. Transmission rates were estimated using a calibration process, detailed in the Appendix, in which we used assumed rates of contact among the age groups to estimate age-to-age specific transmission rates consistent with the current US distribution of pertussis cases. The calibration, using a maximum likelihood estimation approach, was conducted using Microsoft Solver to find the best fit to US age-specific pertussis incidence rates.

**Figure 2:**
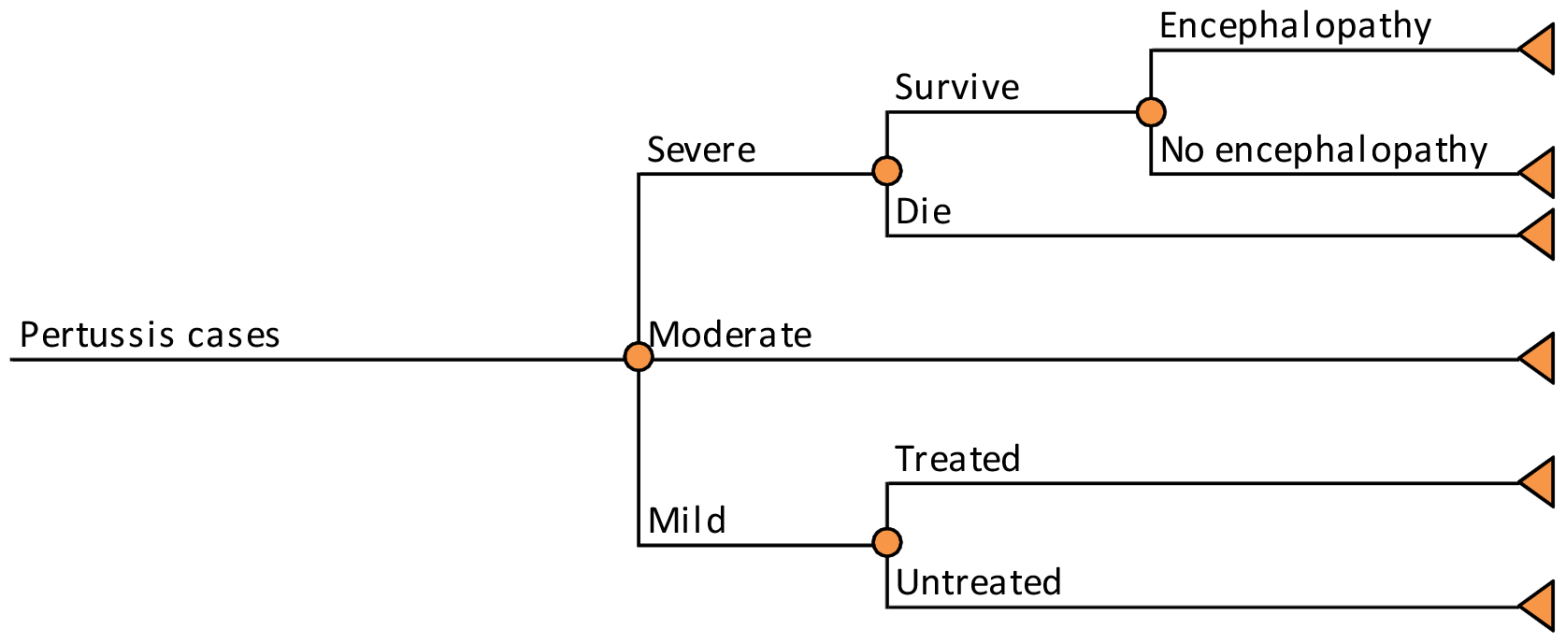
Economic model.

A probability tree was used to estimate clinical outcomes and costs associated with each case of pertussis (Figure 2). Incidence rates derived for each vaccination strategy from the transmission model were applied to the 2010 US population and used as inputs for the economic assessment. The economic analysis was age-stratified into 5 groups: infants (aged <1 year), children (1–9 years), adolescents (10–19 years), and adults (stratified as 20–64 and ≥65 years). Each pertussis case may be mild, moderate, or severe. Only a portion of mild cases will be treated and incur medical and non-medical costs (in addition to the loss of quality-of-life). Severe cases may lead to encephalopathy and/or death; other sequelae of pertussis such as pneumonia were not explicitly modeled, but were accounted for in cost and utility estimates for severe cases. Direct medical and non-medical costs and quality-adjusted life-years (QALYs) lost due to pertussis were assigned to each case based on severity.

### Epidemiologic and vaccine efficacy inputs

DTaP vaccine efficacy was derived from prescribing information for the GSK DTaP vaccine (*Infanrix*®, GlaxoSmithKline) (Table 1) [19]. In the base case, Tdap vaccine was estimated to be 89% effective based on pertussis immunogenicity licensing criteria in a vaccine efficacy trial of DTaP [20]. An alternative scenario analysis was conducted assuming 77% efficacy based on the lower limit of the 95% confidence interval in the DTaP vaccine efficacy trial [20]. Waning of vaccine efficacy is assumed to follow an exponential distribution with a mean duration of protection of 8 years (median 5.5 years) [14]. Sensitivity analyses were conducted with alternative mean duration of protection estimates of 6 and 2 years. For simplicity, DTaP infant vaccine protection was assumed to take effect at 7 months of age.

**Table 1:** Parameters in the Transmission Model.

Parameter	Base-case Value [values used in sensitivity analyses]
Vaccine efficacy	
DTaP	89.0%
Tdap	89.0% [77.0%]
Proportion born with maternal antibodies	26%
Mean duration of protection due to maternal antibodies	3 months
Mean duration of protection due to vaccination or infection:	8 years [6], [10]
Mean duration of infectiousness	
Primary	4 weeks
Recidive	3 weeks
Vaccine Coverage:	
DTaP, doses 1–4, at 1 year	95.0%
DTaP, dose 5, at 5 years	84.4%
Tdap, at 11 years	68.7%
Tdap, at 18–64 years, annual among unboosted	1.7%
Tdap, at 65 years	10.0% [5.0%]
Incidence by age (rate per 100,000)	
<1 yr	435.00
1–6 yrs	61.80
7–9 yrs	67.30
10–18 yrs	49.00
19–64 yrs	124.15
≥65 yrs	86.08

In the model, individuals are protected from pertussis either by maternal antibodies (for infants), acquisition of disease, or vaccination; the model assumes no difference between duration of protection from vaccination and from infection. The proportion of infants with maternal antibodies and the duration of protection from each source were estimated from published and publicly available studies [14], [21]–[23] and clinical opinion. Duration of infectiousness was assumed to differ by type of infection (primary versus recidive) [14], [16].

DTaP and Tdap vaccine coverage for those aged <65 years were estimated from 2010 National Health Interview Survey (NHIS) data. [24]–[26]. For the intervention strategy, we assumed that 10% of the total population of adults aged 65 years receives Tdap. This is consistent with the assumption that 10% of the population aged 65 years is eligible for the decennial Td booster, and 100% of this group receives Tdap; in sensitivity analyses, we halved this number. Because the analysis is performed at equilibrium, 10% of individuals in each age stratum >65 years are assumed to have been vaccinated at age 65, and retain residual vaccine protection consistent with waning assumptions.

Incidence rates used for model calibration were based on 2010 California Department of Public Health surveillance data and 2009 Centers for Disease Control and Prevention (CDC) data [8], [27]. Incidence rates for infants and children <18 were estimated directly from the California data, using the reported incidence among infants <6 months (435 cases/100,000) as the estimated incidence for all infants. For adults 65 years and older, we inflated the CDC 2009 data, assuming 1% reporting, consistent with CDC assumptions [28]. To estimate the incidence for adults aged 18–64, the adjusted incidence for adults aged ≥65 years was multiplied by the ratio of California incidence among adults 18–64 to California incidence among adults aged ≥65 years. Incidence rates were varied in sensitivity analyses, by adjusting the incidence of disease among adults aged ≥65 years from 25–200 cases per 100,000 in fixed increments, while the incidence among younger adults was increased or decreased as a fixed proportion of the ≥65 incidence and pediatric incidence was unchanged. Analyses were also conducted by averaging the California incidence among infants aged <6 months and children 6 months to 6 years of age (61.8 cases/100,000), for a mean of 248 cases/100,000 [8].

Age-to-age specific contact rates used in the transmission model were based on mean daily time of exposure between individuals by age group in the US from 1987–2003 [29]; the contact matrix indicates that most contacts occur between persons of similar age and between parents and children. An alternative contact matrix with European estimates of number of daily contacts was used in sensitivity analyses [30].

### Economic inputs

Cases generated by the model were stratified by age: <1, 1–9, 10–19, 20–64, and ≥65 years old. We further classified each case as severe (requiring hospitalization), moderate (non-hospitalized cases with symptoms of paroxysmal cough), or mild (no paroxysmal cough) as shown in Figure 2. The age-specific proportions of mild, moderate, and severe cases were estimated based on published sources [31], [32].

The cost of treating a mild case was estimated as the cost of antibiotics [33], [34] and a physician visit [35]. The cost of treating a moderate case was based on a prospective study of pertussis morbidity among families in a community setting; costs include physician visits, emergency room (ER) visits, laboratory, and pharmacy costs [36]. The cost of treating a severe case of pertussis was based on total hospitalization cost for pertussis hospitalizations [37].

Patients with severe pertussis are at risk of encephalopathy or death. The probabilities of encephalopathy or death among severe cases were based on 1997–2000 data for reported hospitalized cases [29]. We used the probability of encephalopathy to estimate loss of quality-of-life associated with long-term disability in addition to the loss of quality-adjusted life-years due to death. We estimated that 33% of encephalitis cases would result in permanent sequelae, characteristic of encephalopathy [29]. Because the acute cost of encephalopathy was assumed to be included in the hospitalization cost, it was not estimated separately; long-term costs of encephalopathy were not considered. A scenario assuming no cases of encephalopathy was also included as a sensitivity analysis. Due to limited data on treatment of mild pertussis, we assumed that the proportion of mild pertussis cases seeking treatment is the same as the proportion of influenza patients receiving outpatient care [38].

Costs were derived from published and publicly available sources and were converted to 2010 U.S. dollars, as necessary, using the medical component of the Consumer Price Index [39]. Vaccine price was obtained from the CDC vaccine price list [40] and the total vaccination cost includes cost of vaccine-related adverse events [16]. For sensitivity analyses, the lower public sector vaccine cost estimated from the CDC vaccine price list was used. We calculated the annual cost of the vaccination program by multiplying the unit vaccine cost by 10% of the number of US residents aged 65 years.

Direct non-medical costs include lost productivity from hospitalization due to time missed from work for hospitalized adults and adolescents who are employed, and caregiver time for acute illness and hospitalization in infants, children, and adolescents. Age-specific work loss due to hospitalization was applied to severe cases only. We estimated lost productivity based on published estimates of hospital length of stay combined with estimates of the age group-specific employment rates [14], [16], [41], [42]. Lost productivity for caregivers of children was calculated based on the wage rate for adults aged 20–64 years. We assumed no caregiver time loss for adult cases (including adults aged 65 years and older).

Utility values, preference-based measures of quality-of-life ranging from 0 to 1 used to calculate QALYs, were obtained from published literature [14], [41], [43]. Disutilities for mild, moderate, and severe pertussis and encephalopathy were used to estimate the quality of life loss associated with pertussis. Disutilities associated with acute disease symptoms were assigned to pertussis cases for lengths of time that differed by severity and age, based on duration of mild, moderate, and severe symptoms. Total duration of illness, obtained from published literature [14], was assumed to be the same for severe, moderate, and mild disease (Table 2). A mild case was assumed to incur the mild symptom disutility for the full length of illness. Moderate adolescent/adult cases receive two weeks of moderate symptom disutility followed by mild symptoms disutility for the remaining days of illness. A severe adolescent/adult case incurs two weeks of severe symptom disutility followed by mild symptoms disutility for the remaining days of illness. Similarly, infants are assumed to incur 4 weeks of moderate or severe symptoms disutility followed by mild, and children incur 3 weeks of moderate or severe symptoms disutility followed by mild. Sensitivity analyses were conducted with reduced duration of illness and reduced duration of the most severe symptoms.

**Table 2:** Parameters in the Economic Model.

Parameter	Age Group (years)/Base-case Value [values used in sensitivity analyses]				
	<1	1–9	10–19	20–64	≥65
**Population**	310,751,161				
Population distribution	1.39%	12.27%	13.24%	60.03%	13.06%
**Clinical**					
Proportion of Severe, Moderate, and Mild Cases*					
% mild	82.1%	21.2%	24.3%	11.0%	14.0%
% moderate	7.4%	73.3%	73.6%	86.0%	74.0%
% severe	10.5%	5.5%	2.1%	3.0%	12.0%
% with encephalopathy (permanent sequelae) among severe cases	0.32%	0.71%	2.38%	1.43%	1.43%
% dying among severe cases	1.18%	0.71%	0.00%	0.86%	0.86%
% mild cases that receive medical care	47.1%	40.5%	35.2%	37.2%	70.7%
Duration of symptoms	80 days [56]	56 days	74 days [56]	87 days [56]	87 days [56]
**Costs**					
Vaccine Cost	NA	NA	NA	NA	$18.10 [$16.16, $17.84]
Cost of Treating Pertussis, by Severity:					
Mild	$82.80	$93.00	$101.66	$99.22	$99.22
Moderate	$607.21	$322.83	$268.83	$204.72	$203.13
Severe	$13,029.47	$6,431.77	$7,066.87	$7,221.97	$7,221.97
Days of work lost due to acute illness incurred by caregiver (all treated cases)	2.1	2.1	0.8	-	-
Days of work lost due to hospitalization (severe only)					
By caregiver	2.1	1.3	1.1	-	-
By self	-	-	0.3	2.4	0.6
Value of lost productivity per day	NA†	NA†	$52.38	$157.45	$114.30
**Disutility**					
Pertussis symptoms:					
Mild	0.15 [0.14,0.12]	0.18 [0.17,0.15]	0.14 [0.13,0.11]	0.1 [0.09,0.08]	0.1 [0.09,0.08]
Moderate	0.15 [0.14,0.12]	0.26 [0.23,0.21]	0.21 [0.18,0.16]	0.15 [0.14,0.12]	0.15 [0.14,0.12]
Severe	0.42 [0.38,0.34]	0.37 [0.33,0.30]	0.3 [0.27,0.24]	0.19 [0.17,0.15]	0.19 [0.17,0.15]
Encephalopathy	0.20 [0.18,0.16]	0.20 [0.18,0.16]	0.20 [0.18,0.16]	0.20 [0.18,0.16]	0.20 [0.18,0.16]

The number of cases that are mild among children less than 10 years old was based on the proportion of unreported cases, while those in individuals 10 years and older was based on the percentage of reported cases without paroxysmal cough [10], [31]. The proportion of cases that are severe was based on hospitalization rates [10], [32]. In children less than 10 years old, hospitalization rates were only applied to reported cases. The proportion of cases that are moderate was assumed to be those not classified as severe or mild.

Lost productivity per day for children <10 years old was assumed to be incurred by adults 20–64 years old.

In addition to disutility of acute illness, patients with encephalopathy are assumed to incur the disutility for encephalopathy for the remainder of their lifetime. Loss of QALYs due to death was based on age-specific US population norms [44]. Total QALYs lost due to death or encephalopathy were calculated as the discounted present values of the lost utility streams over the remaining expected lifetime, based on age-specific life expectancy [45] at the time of the event. In sensitivity analyses, we explored how results change if reductions in utility are 20% smaller than the base-case value.

### Analyses

Outcomes were assessed for the entire US population, including children and adults outside the target ≥65 age group, to capture the indirect benefits of vaccination in the unvaccinated population. The epidemiologic model uses a time step of 1/1200 year, or approximately 7 hours, to simulate continuous movement between compartments. The transmission model was calibrated and run assuming 1000 individuals in each one-year age group to calculate disease rates. The model estimates age-specific incidence rates of primary and recidive infection; these rates are then projected over the US population to estimate pertussis incidence under both the baseline and intervention strategies. When linked with the epidemiologic model, the economic model projects costs and QALYs associated with vaccination and pertussis. Cost measures include direct medical costs, (costs of vaccination and costs of treating pertussis cases), and direct non-medical costs (lost productivity from hospitalization and caregiver time) for the societal perspective. The payer perspective excludes direct non-medical costs. The model estimates cases avoided stratified by severity, deaths avoided, encephalopathy avoided, and, where applicable, incremental cost-effectiveness ratios (ICER) in terms of incremental cost/QALY gained. Costs and utility reductions associated with acute illness are assumed to occur within the one-year time horizon and therefore are not discounted. Lifetime QALYs lost due to death and encephalopathy are discounted at 3% per year back to the base year in which the event occurs, consistent with US recommendations [46].

### Sensitivity analyses

To assess the sensitivity of the model results to manipulation of influential parameters, a series of deterministic sensitivity analyses were conducted, by varying incidence, duration of protection, vaccine efficacy, vaccine coverage, vaccine cost, as well as direct medical and non-medical costs using a previously published cost-effectiveness study as an alternative secondary source [16]. Additional scenario analyses were conducted in which we used a European contact matrix [31], assumed 0% encephalopathy, decreased utility losses, and reduced the duration of the most severe pertussis symptoms. In addition, we replicated assumptions from a previously-published economic analysis [14], assuming the same structure and transmission parameter estimates, to explore the impact of including asymptomatic infections in the disease model.

## Results

Under both the baseline and intervention strategies, the model predicts that the one-year pertussis incidence will be greatest among infants, followed by adults aged 20–64 years, adults aged ≥65 years, children, and adolescents (results not shown). Incidence rates of pertussis infection among infants, children, adolescents, and adults aged 20–64 years are predicted to decrease by approximately 28–29% with the intervention strategy, whereas in adults aged ≥65 years, the yearly incidence rate is predicted to decrease by 34% (results not shown). The numbers of cases avoided by the intervention strategy, by age group, is reported in Table 3. The greatest number of cases avoided (>66,000) is observed among adults aged 20–64 years, although when adjusted for the size of the affected population, the impact is greatest among infants. For the US population of 311 million, the intervention strategy is expected to prevent 4,188 severe cases, 72,506 moderate cases, and 20,357 mild cases of pertussis per year at steady state.

**Table 3:** Cases Avoided with Intervention Strategy, by Age Group, Case Severity, and Treatment.

	Age Group (years)					
Case Type	<1	1–9	10–19	20–64	≥65	All ages
Severe cases	525	109	140	1,987	1,427	4,188
Deaths	6.2	0.8	0.0	17.0	12.2	36.2
Encephalopathy	1.7	0.8	3.3	28.1	20.2	54.2
Moderate Cases	372	1,454	4,921	56,961	8,798	72,506
Mild Cases	4,458	5,323	1,626	7,286	1,664	20,357
Treated	2,100	2,159	573	2,707	1,176	8,715
Untreated	2357	3165	1053	4579	488	11642
Total Cases	5,355	6,886	6,687	66,234	11,889	97,051

Introduction of the intervention strategy is expected to lead to yearly direct medical cost savings of approximately $49.5 million. From the societal perspective, cost savings related to acute pertussis infection and outcomes increase to $52.4 million per year (Table 4, top panel). The additional total yearly cost of vaccinating all individuals aged 65 years under the intervention versus baseline strategy is estimated to be $4.7 million (Table 4, middle panel). The net cost savings including the cost of the vaccination program are $44.8 million from the payer perspective, and $47.7 million from the societal perspective (Table 4, bottom panel). In the base case, the intervention strategy dominates the baseline strategy by being both less costly and more effective (by >3,000 QALYs) than the baseline strategy (Table 5).

**Table 4:** Incremental Costs of Intervention Strategy.

	Age Group (years)					
Cost Item	<1	1–9	10–19	20–64	≥65	All ages
***Costs (Savings)***						
Mild treated	($173,902)	($200,740)	($58,250)	($268,594)	($116,697)	($818,183)
Moderate	($226,103)	($469,358)	($1,322,867)	($11,660,750)	($1,787,022)	($15,466,101)
Severe	($6,839,132)	($703,840)	($992,437)	($14,350,102)	($10,303,145)	($33,188,657)
**Direct Medical**	($7,239,138)	($1,373,939)	($2,373,554)	($26,279,446)	($12,206,864)	($49,472,941)
Lost productivity	($1,183,730)	($806,503)	($121,473)	($749,418)	($89,816)	($2,950,939)
**Direct Medical and Non-medical**	($8,422,867)	($2,180,441)	($2,495,027)	($27,028,864)	($12,296,680)	($52,423,880)
***Vaccination Costs***						
Incremental vaccination costs	$0	$0	$0	$0	$4,691,839	$4,691,839
***Net Costs (Savings)***						
Excluding productivity loss	($1,580,476)	($1,339,145)	($336,222)	($55,113,000)	($7,515,026)	($44,781,102)
Including productivity loss	($8,422,867)	($2,180,441)	($2,495,027)	($27,028,864)	($7,604,842)	($47,732,041)

**Table 5:** Base-case Results.

Societal perspective (including lost productivity)					
Comparator	Total Costs	QALYs lost	Incremental Cost	Incremental QALYs	ICER
Baseline strategy	$238,884,106	11,614	–	–	Reference
Intervention strategy	$191,152,065	8,227	−$47,732,041	3,387	Dominant

Payer perspective (excluding lost productivity)					
Baseline strategy	$228,571,260	11,614	–	–	Reference
Intervention strategy	$183,790,158	8,227	−$44,781,102	3,387	Dominant

### Sensitivity analyses

**Figure 3:**
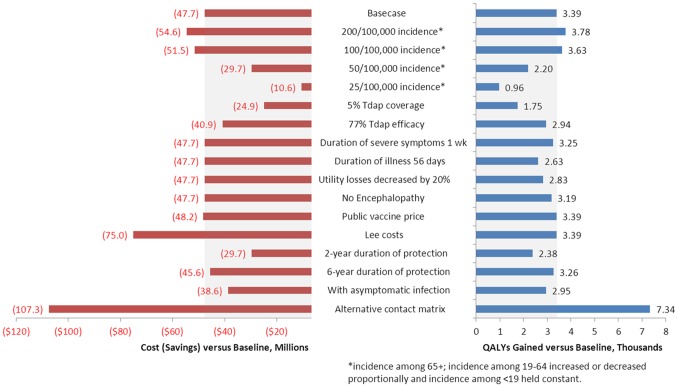
Sensitivity and scenario analyses varying selected parameters.

Increasing the incidence of pertussis from the base case of 86.6 cases per 10,000 in those aged ≥65 years to 200 cases per 100,000 results in a $6.8 million increase in cost savings and nearly 400 more incremental QALYs gained (societal perspective) for the intervention strategy (Figure 3). Reducing incidence has the opposite effect of reducing cost savings and QALYs gained; the Tdap vaccination program nevertheless remains dominant even at incidence as low as 25 cases per 100,000 in those aged ≥65 years. As expected, decreasing vaccine efficacy and coverage and accelerating waning of vaccine protection results in lower cost savings and fewer QALYs gained. Likewise, alternative assumptions that reduce the impact of disease, including removing encephalopathy from the model, decreasing utility losses associated with disease, decreasing the duration of illness and decreasing the duration of the most severe symptoms, all result in fewer cost savings and/or QALYs gained. Assuming lower vaccine costs using public pricing increases cost savings by $500,000 per year while using the alternative source for costs increases cost savings by $27 million. Including asymptomatic infection in the model results in approximately 430 fewer QALYs gained and decreases cost savings by >$6 million because the natural protection provided by asymptomatic infection reduces the potential benefit of the vaccine. Recalibrating and rerunning the model using the alternative European contact matrix results in >100,000 more cases prevented and almost $60 million more in cost savings; this appears due to greater contact between those aged ≥65 years and both infants and adolescents in Europe. Under all scenarios examined, the intervention strategy remains dominant (less costly and more effective than the baseline strategy) from both the payer and societal perspectives.

## Discussion

We constructed a population-level disease-transmission model to assess the cost effectiveness of replacing the decennial Td booster with Tdap in adults aged 65 compared to baseline practice prior to 2010 of no Tdap vaccination in this age group. Model results indicate that administering Tdap booster vaccination to 10% of eligible adults at the age of 65 years would prevent 97,000 cases of pertussis each year (>5,300 in infants), including 4,200 severe cases (>500 in infants). Vaccinating eligible adults aged 65 years is cost-saving, providing annual net societal cost savings of $44.7 million and $47.7 million, from the payer and societal perspectives, respectively. In the base case as well as all sensitivity analyses, the strategy of adding Tdap vaccination in the adults aged 65 years to the baseline strategy is dominant compared to the baseline strategy, as it is both less costly and more effective than the baseline strategy.

Although no other published models have specifically examined the cost-effectiveness of vaccinating the US population aged ≥65 years with Tdap, our findings are generally consistent with a recent analysis by Coudeville and colleagues in which they evaluated the cost-effectiveness of adult vaccination in the presence of infant and adolescent vaccination using a dynamic transmission model. They found strategies of adding one-time adult boosting at age 40 with cocooning, and repeated decennial adult vaccination, both to be dominant versus the baseline strategy of infant plus adolescent vaccination in terms of life-years saved [15]. Although Coudeville found the optimal age at boosting to be 40 years, given the low coverage in this age group, the strategy we examine of vaccinating adults aged ≥65 years may be an effective approach to increasing overall adult coverage.

Our finding that Tdap vaccination in adults aged 65 years is cost-saving can be compared to other studies of vaccination in this population. For example, a strategy of universal influenza vaccination including vaccination of those aged ≥65 years was similarly found to be cost-saving [47]. In contrast, a recent analysis of replacement of the 23-valent polysaccharide pneumococcal vaccine with the 13-valent conjugate vaccine in routine vaccination at ages ≥65 years found this switch to be more costly, but cost-effective at $28 900 per QALY gained [48]. Similarly, vaccinating adults aged >60 years for herpes zoster was estimated to cost <$50,000 per QALY gained [49]. These studies did not consider indirect effects in the unvaccinated populations, and results may have been more favorable had these effects been taken into account.

The findings of our study are based on a number of assumptions, and limitations of the model should be considered when interpreting results. The transmission model structure is a simplified representation of disease transmission and outcomes of pertussis. The possible transitions between health states in the model are based on our current understanding of pertussis epidemiology, but may be subject to revision as new information becomes available. In addition, although we accounted for differences in treatment and outcomes in the model using stratification by age and disease severity, we recognize that the diversity of the US population and the complexity of the health-care delivery system may not be well represented by the relatively simple structure of this model. Furthermore, this model is not structured to capture family or household dynamics that would allow for evaluation of cocooning related to vaccination of those aged ≥65 years. To evaluate this effect, the model would have to account for grandparents' higher likelihood of both interacting with grandchildren and of vaccination, whereas our model treats those aged ≥65 years as a homogeneous population. In addition, because younger adults interact with both infants and grandparents, if we had assumed vaccination uptake in this age group to be weighted toward parents and caregivers this also may have affected model results. Because cocooning targets vaccination to those most likely to transmit disease to infants, a model including cocooning likely would have predicted more infant disease prevented with the ≥65 Tdap vaccination program.

We also note that our model structure does not take into account other epidemiologic phenomena related to pertussis incidence, including the possible loss of boosting of pertussis immunity with lower incidence of disease, or the possible replacement of the vaccine type strains by more virulent strains of pertussis [50]. Modeling these phenomena would require a more complex model than that described in this report, as well as greater understanding of the disease dynamics of pertussis.

In addition, the transmission model as designed, evaluates the intervention vaccination program versus the baseline vaccination program assuming both are at equilibrium; however, recent pertussis incidence data have shown a pattern of decreased incidence following the 2005 adult and adolescent vaccination recommendation, followed by an apparent increasing trend in incidence thereafter. Because the reason for this recent pattern in incidence is unclear and may represent changes in reporting rather than in the true incidence, we chose to assume that incidence is neither increasing nor decreasing for our analyses.

A further potential limitation of the steady-state analysis is that it does not take into account the difference in timing between vaccine costs and benefits accruing from the program. The current-year benefits of the pertussis vaccination program are due not only to the protection provided to individuals vaccinated in the current year but also to those vaccinated in prior years whose protection is waning. At the same time, the one-year model does not capture the full benefits of the current year's vaccination program, as many of these benefits will be realized in the future, beyond the model time horizon. At steady state, we may assume that any benefits of past vaccination being counted in the current year are perfectly offset by the uncounted future benefits of the current year's vaccination program. However, this assumption disregards the vaccine costs incurred at the start of the vaccination program before equilibrium was achieved, as the decrease in cases with the fully-implemented program at steady state is due, in part, to changes in disease epidemiology that likely took several years of vaccination to achieve. To investigate the change in disease incidence over time, we ran the model to steady state and found that the yearly incidence indeed declined slowly, with the initial post-vaccination years experiencing only modest declines in incidence, and the herd effect building over time.

In addition to the structural limitations in the model design, we note that data used to estimate model inputs were derived and synthesized from a variety of sources; this process is subject to bias and different assumptions may have yielded different results. In particular, recent outbreaks and supporting epidemiologic data have called into question the effectiveness and durability of protection from vaccination with acellular pertussis vaccine [7], [9]. Published estimates from California, suggest that the acellular pertussis vaccine may wane to 47% of its original efficacy after 5 years (i.e., from 90% to 42% efficacy) [9]. Assuming a constant waning rate, this suggests a mean time to waning of 6.6 years; sensitivity analyses demonstrate that even assuming this shorter period of effectiveness, the vaccine would remain cost-saving in our model. We further found that even under very unfavorable assumptions in which the protection afforded by pertussis infection is assumed to be 8 years and that provided by vaccination 2 years, the 65+ vaccination program is expected to save $2.7 million.

Because of underreporting of pertussis to US surveillance systems, the true incidence of pertussis is unknown and was estimated by combining data sources and inflating CDC estimates. The data and assumptions used to derive these estimates could not be externally verified and may have provided over- or under-estimates of the true incidence across age groups. Moreover, by calibrating the model to 2010 incidence data in the transmission model, collected only 5 years after the Tdap vaccination was expanded to adolescents and adults, we are potentially overestimating pertussis incidence at steady state. However, even in sensitivity analyses in which we scaled the incidence estimates from the basecase of 86 per 100,000 among those ≥65 to 50 and 25 per 100,000, Tdap remained cost saving with estimated savings of $29.7 and $10.6, million respectively, from the societal perspective.

We further note that our assumption that 10% of those aged 65 would be vaccinated per year may be optimistic, given current US vaccination rates for TD (54.4% report receiving in the last ten years) and pneumococcal vaccines (62.3% report receiving ever) in the ≥65 age group [51]. However, sensitivity analyses showed that even if only 5% of those aged 65 are vaccinated per year, the vaccine remains cost saving, and provides substantial benefits. Sensitivity and scenario analyses, in which we varied inputs for incidence, contact matrix, age of DTaP protection, waning rates, vaccine efficacy, vaccine coverage, vaccine cost, direct medical and indirect costs, and discounting, show the intervention vaccination program remains cost saving under all scenarios examined, with savings ranging from approximately $100,000 to $10 million compared to the baseline vaccination strategy.

In conclusion, our study found the program of replacing the decennial Td booster with Tdap among US residents aged 65 years is projected to prevent 97,000 cases of pertussis per year, and is cost-saving from societal and payer perspectives.

*Infanrix®* is a trademark of the GlaxoSmithKline group of companies.

## Supporting Information

Appendix Figure S1 **Model-predicted Versus Target Pertussis Incidence after Model Calibration – 35 years.**
(TIF)

Appendix S1 **Comparison of model-predicted and observed incidence, by age, after model calibration.**
(DOCX)
