# Supplementary figures and images for: Cost-Effectiveness of Tdap Vaccination of Adults Aged ≥65 Years in the Prevention of Pertussis in the US: A Dynamic Model of Disease Transmission
Source: PLoS One. 2014 Jan 9;9(1):e72723. doi: 10.1371/journal.pone.0072723 (PMC3886978; doi:10.1371/journal.pone.0072723)

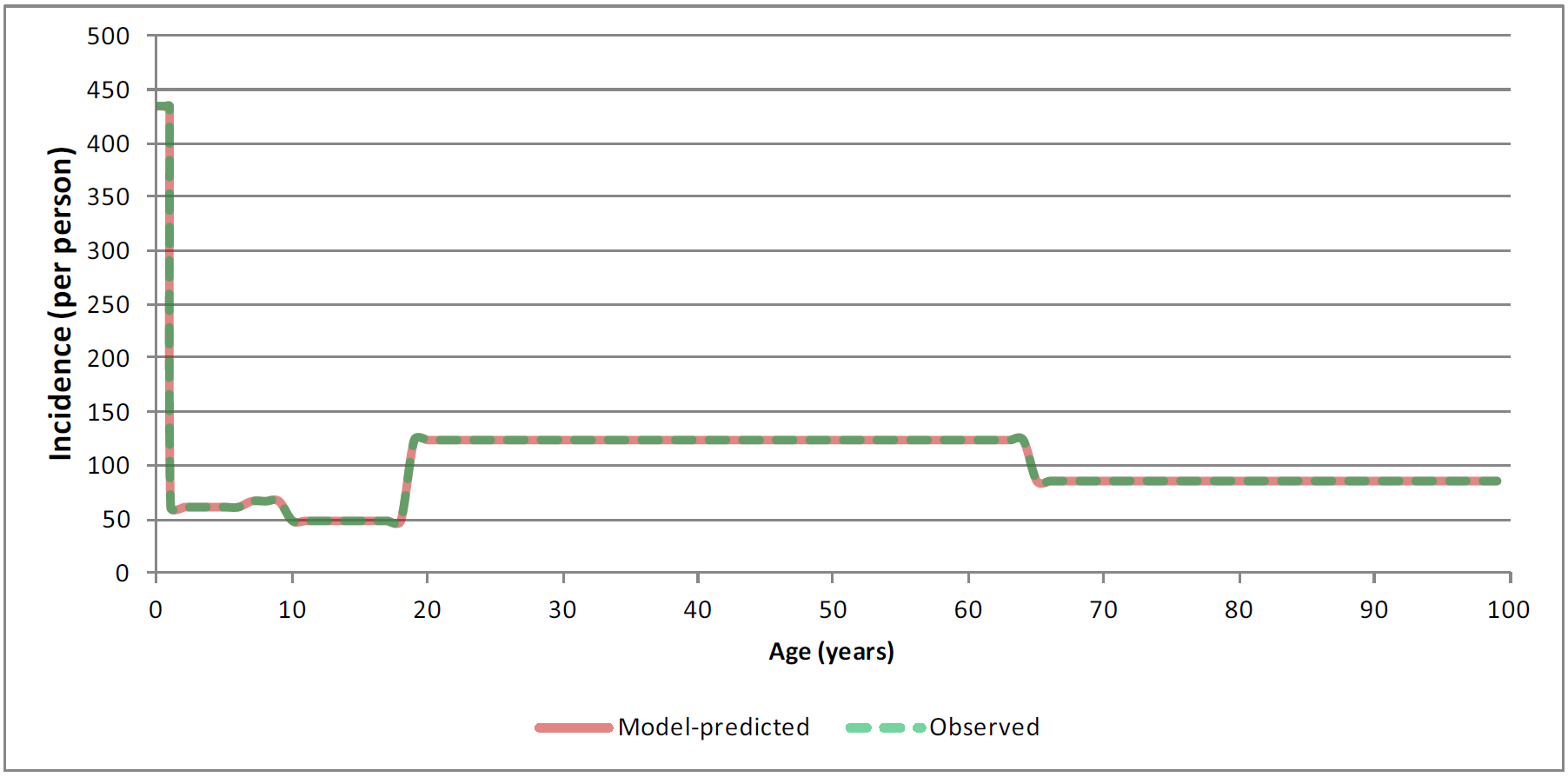

Supplement: Appendix Figure S1 — Model-predicted Versus Target Pertussis Incidence after Model Calibration – 35 years. (TIF) [file pone.0072723.s001.tif]
