# Supplementary material for: Cost-Effectiveness of Tdap Vaccination of Adults Aged ≥65 Years in the Prevention of Pertussis in the US: A Dynamic Model of Disease Transmission
Source: PLoS One. 2014 Jan 9;9(1):e72723. doi: 10.1371/journal.pone.0072723 (PMC3886978; doi:10.1371/journal.pone.0072723)
Supplement: Appendix S1 — Comparison of model-predicted and observed incidence, by age, after model calibration. (DOCX) [file pone.0072723.s002.docx]

**Appendix**

The transmission model was calibrated to estimate the force of infection (λ), transmission co-efficient (β), and the starting compartment distribution of the population.

Force of infection (λ) is the rate at which susceptible individuals become infected by pertussis and is described by the following equation:

λ(a,t)=∫ c(a,a’) [β_1_I_1_(a’,t)+ β_2_I_2_(a’,t)] da’

where λ is the force of infection in a susceptible individual in age-group *a* at time *t*;c is the duration of contact between a susceptible individual in age-group *a* with an infected individual in age-group *a’*;β is the transmission co-efficient or the probability that contact (c) between a susceptible and infected individual leads to transmission of disease by type of infection (β1 orβ2); and I is the prevalence of infected individuals in age-group *a’* at time *t* by type of infection (I1 or I2). Each type of infection is associated with a different prevalence and set of transmission coefficients. The ratio between the transmission coefficients for I1:I2 is assumed to be 1:0.7 (i.e., I2 patients have a 70% chance of transmitting disease relative to I1 patients).[14]

The model was calibrated to steady state by solving all model equations at equilibrium. The calibration was run assuming 1000 individuals in each year of age. Inputs for the calibration include population size, duration of protection, duration of infectiousness, vaccine efficacy, vaccine coverage, and the age-group to age-group contact matrix. The calibration targets are the age-specific incidence of pertussis presented in Table 1.

When calibrated at equilibrium, the transmission model generated incidence estimates that closely matched the calibration targets (Figure ).

After introduction of new vaccination, the model is run forward 35 years to achieve equilibrium under the new scenario. Disease incidence and the distribution of patients by disease/vaccination status remained near constant after 35 years; the total number of cases from year 34 to 35 decreased by 0.061%.

**Figure legend: Comparison of model-predicted and observed incidence, by age, after model calibration**
